# A description of sleep behaviour in healthy late pregnancy, and the accuracy of self-reports

**DOI:** 10.1186/s12884-016-0905-0

**Published:** 2016-05-18

**Authors:** Jordan P. R. McIntyre, Cayley M. Ingham, B. Lynne Hutchinson, John M. D. Thompson, Lesley M. McCowan, Peter R. Stone, Andrew G. Veale, Robin Cronin, Alistair W Stewart, Kevin M. Ellyett, Edwin A. Mitchell

**Affiliations:** Department of Obstetrics and Gynaecology, University of Auckland, Auckland, New Zealand; New Zealand Respiratory and Sleep Institute, Auckland, New Zealand; Department of Paediatrics, Child and Youth Health, University of Auckland, Auckland, New Zealand; Department of Epidemiology and Biostatistics, School of Population Health, University of Auckland, Auckland, New Zealand; Respiratory Measurement Laboratory, Auckland District Health Board, Auckland, New Zealand

**Keywords:** Pregnancy, Self-report, Questionnaire, Sleep study, Polysomnography

## Abstract

**Background:**

The importance of maternal sleep and its contribution to maternal and fetal health during pregnancy is increasingly being recognised. However, the ability to accurately recall sleep practices during pregnancy has been questioned. The aim of this study is to test the accuracy of recall of normal sleep practices in late pregnancy.

**Methods:**

Thirty healthy women between 35 and 38 weeks of gestation underwent level III respiratory polysomnography (PSG) with infrared digital video recordings in their own homes. Data regarding sleep positions, number of times getting out of bed during the night and respiratory measures were collected. A sleep questionnaire was administered the morning after the recorded sleep. Continuous data were assessed using Spearman’s Rho and Bland-Altman. Cohen’s Kappa was used to assess recall in the categorical variables.

**Results:**

Two-thirds of participants went to sleep on their left side. There was good agreement in sleep onset position between video and questionnaire data (Kappa 0.52), however the there was poor agreement on position on wakening (Kappa 0.24). The number of times getting out of bed during the night was accurately recalled (Kappa 0.65). Twenty five out of 30 participants snored as recorded by PSG. Questionnaire data was inaccurate for this measure. Bland-Altman plots demonstrated acceptable agreement between video and questionnaire data for estimated sleep duration, but not the time taken to fall asleep (sleep latency). One participant had mild obstructive sleep apnoea and another probable high upper airways resistance.

**Conclusions:**

Sleep onset position, sleep duration and the number of times getting out of bed during the night were accurately recalled, but sleep latency and sleep position on waking were not. This study identifies the sleep variables that can be accurately obtained by questionnaire and those that cannot.

**Electronic supplementary material:**

The online version of this article (doi:10.1186/s12884-016-0905-0) contains supplementary material, which is available to authorized users.

## Background

The importance of maternal sleep in late pregnancy (>28 weeks), in particular sleep position, with respect to stillbirth risk was identified by The Auckland Stillbirth Study [1] and has been confirmed by two subsequent studies [2, 3]. Fatal outcomes related to sleep disordered breathing [4] and supine hypotension syndrome [5] have been documented, demonstrating their potential significance in sleep in pregnancy, and thus the importance of accurate recall. The data reported in The Auckland Stillbirth Study relied on maternal recall of sleep behaviour, a limitation identified by the authors and in an accompanying editorial [6]. The editorial [6] and subsequent correspondence highlighted the possibility of recall bias.

In 1994 Mills et al. [7] observed the sleep of 51 pregnant women (≥30 weeks gestation) and 31 non-pregnant controls in antenatal and gynaecological wards and noted their initial sleep position. The majority of pregnant women settled to sleep in lateral positions, predominantly left lateral (77 %), with only 2 % supine. The non-pregnant controls’ sleep onset positions were more evenly spread amongst the left (26 %), right (32 %) and supine (38 %) positions.

More recently, O’Brien and Warland [8] reported the typical night time sleep positions and duration of position adopted by 51 pregnant women (7–38 weeks gestation, mean 28.3 ± 6.9 weeks) using level III respiratory polysomnography (PSG) sleep studies. In the subgroup analysis of the participants who were greater than 28 weeks gestation (*n*=33), the median proportion of time spent supine (26.5 %) was less than time spent on the left (35.7 %) and right (35.5 %) lateral. The duration spent in each position, sleep onset and awakening positions, and the accuracy of the women’s recall were not reported.

Warland and Dorrian [9] validated the ability of pregnant women to recall sleep position over three nights using video analysis and sleep diaries. They instructed the women to settle to sleep in the left lateral position and to resettle in the left lateral if they woke, and asked them to report how long they believed they spent in that position overall. These two studies are complementary, in that the first describes normal sleep practices [8], and the latter reliability of recall [9].

The Auckland Stillbirth Study identified that self-reported maternal position at sleep onset, waking position and the number of times getting up at night to use the toilet may be associated with likelihood of late stillbirth. However the ability of the women to accurately recall this information was not known. It is therefore pertinent to report the accuracy of recall of night-time sleep, not influenced by investigator instruction.

The purpose of this study was to assess the accuracy of participant recall of normal sleep practices in late pregnancy.

## Methods

### Participants

Healthy women aged ≥ 18 years with a normal singleton pregnancy, late in the third trimester (35–38 weeks gestation), were recruited from low risk midwifery care. Exclusion criteria included: current smoking or alcohol use, any medical or obstetric complications (e.g. intrauterine growth restriction, preeclampsia, any known cardiovascular, respiratory or renal disorders, all forms of diabetes), not regularly attending scheduled obstetric appointments, and orthopaedic or musculoskeletal conditions which would make adopting different maternal positions difficult. Birth outcome data were collected to confirm the normal health status of the mother and neonate.

### Experimental design/protocol

All sleep studies were performed in the participants’ homes. The participants were set-up for Level III respiratory PSG (Embletta® Gold, Embla, Broomfield, CO, USA) and infrared digital video recording of sleep (HDR-SR12E Camcorder, Sony, Tokyo, Japan) with an external infrared light (Sony HVL-IRM). The level III respiratory PSG is a sleep study without electroencephalography (EEG) for sleep staging. The camcorder was set at a high definition 1920 × 1080i resolution and a sample rate of 25 Hz. The PSG devices were programmed to start one hour before the participants’ estimated bed time, and to stop one hour after the estimated wake time. To ensure clock-synchronisation of the sleep apparatus with the video, participants pressed the event marker button in view of the camera immediately before turning off the room lights. Participants were instructed to sleep as normal with regards to bed time and wake time, number of pillows and lighting. However, some bed partners chose not to sleep in the same bed on the study night. Participants abstained from caffeine, chocolate and strenuous exercise on the day of study. Current and pre-pregnancy Body Mass Index (BMI; kg.m^−2^) were calculated using self-reported height and weight measurements. Gestation at the time of assessment was based on first trimester ultrasound scanning.

A sleep questionnaire was administered as a face-to-face interview the morning after the PSG. No information regarding the content of the questionnaire was given to the participants prior to its completion. The participants were told the purpose of the study was to describe normal sleep in healthy pregnancy and were not informed that a purpose of the study was to assess the accuracy of their responses, so as not to influence their awareness of sleep, how they might sleep, and their questionnaire responses. Furthermore, no prompts were given to help them recall any aspect of their sleep for the questionnaire. The questionnaire is available as supplementary material (Additional file 1).

### Data analysis

The questions participants were asked and corresponding video measures are presented in Table 1. This questionnaire was similar to that used in a previous study by our research group [10], itself based on the sleep aspects of the questionnaire portion of The Auckland Stillbirth Study dataset [1, 11]. The additional question about snoring on the study night was compared to measures of snoring on the derived snore trace from the nasal cannula. The automated output snoring measures generated by the sleep software were total snore time, snore time as a proportion of the study period, number of snoring episodes over the study period, mean duration of each snoring episode, and longest snoring episode. Due to the inability to control for degree of incline and head-tilt that the women adopted during sleep (and the difficulty in measuring it accurately), no distinction was made between supine and semi-recumbent positions, with all marked as “supine”.

**Table 1:** Questionnaire and video comparisons

Questionnaire	Video
Sleep latency: How long do you think you took to fall asleep?	Time from first time recumbent until first completed three minutes period of immobility.
Sleep duration: How many hours of actual sleep do you think you got?	Time between first time asleep (as estimated by above criteria) and last time recumbent, minus time spent out of bed.
If you woke up, how many times did you go to the toilet?	Number of times observed getting out of bed.
What position did you fall asleep in last night?	First position with greater than three minutes immobility.
What position did you wake up in?	Last position with greater than three minutes immobility.
Did you change sleep position during the night? 0: Not at all 1: Possibly once 2: Possibly twice 3: More than twice but not lots 4: Lots of times.	Number of positions greater than three minutes. 0: No changes 1: One change 2: Two changes 3: Between three and eight changes^a^ 4: Nine or more changes^a^.
Did your legs twitch or jerk often while you slept last night? Yes, No, Don’t know	Presence or absence of sustained leg movements not associated with position changes.

^a^determined based on distribution of questionnaire responses

The study duration was calculated as the time interval from the first to last recumbent position observed in the video. As there are no clearly defined criteria for defining sleep onset from video data, it was defined as the first three minute period with no movements, similar to that used previously in an accelerometry study [12]. Position changes were therefore counted as positions lasting three minutes or longer, with shorter duration positions considered to be part of the transition between sleep positions. Sleep latency (time taken to fall asleep), sleep duration, and toilet visits (questionnaire) or getting out of bed (video), were assessed for agreement between video and questionnaire by Spearman’s Rho. Bland-Altman plots were also used to assess pregnant participants’ recall of sleep latency and sleep duration. Cohen’s Kappa was used to assess recall in the categorical variables. In addition to the questionnaire responses compared to video data in Table 1, additional questions were asked to describe normal sleep. Participants were asked if they had difficulty getting to sleep (Yes, No) and getting back to sleep if they woke (Yes, No, Didn’t wake), if they were restless on the night of the study (Not at all, A little, Average, More than average, Very restless), and their overall sleep quality on the study night (Very good, Fairly good, Average, Fairly bad, Very bad). Unless otherwise stated, continuous sleep data are presented as Median (Range). All PSG data were assessed by a single observer (JM) according to the American Academy of Sleep Medicine 2012 guidelines [13]. All video data were assessed by a single observer (CI). Statistical analyses were performed using SPSS Statistics version 22 (IBM Corp., Armonk, NY).

The data presented here were collected concurrently as part of a study of polysomnography during pregnancy. The sample size was thus primarily calculated according to the physiological outcomes for that study and not for the accuracy of the questionnaire responses addressed in this manuscript.

## Results

### Participants

Thirty pregnant participants completed this study. Twenty four were in their first (ongoing ≥20 weeks’) pregnancy and six had had one or more previous births. The mean gestation at assessment of 37 weeks’, was normally distributed. Birth outcome data were available for 29 participants and birth weights for 27 participants; the remaining participant could not be contacted. Mean gestation at birth was 40 ± 1 weeks’ (*n*=29) and mean birth weight 3410 ± 391 g (*n*=27). All infants were live born, and free of congenital abnormalities (*n*=29). Participant characteristics are presented in Table 2.

**Table 2:** Participant characteristics; Mean ± standard deviation

Age (years)	30.8 ± 5.2
Height (cm)	165.4 ± 6.6
Current Weight (kg)	76.8 ± 14.1
Pre-pregnancy weight (kg)	62.8 ± 12.3
Current BMI (kg.m^−2^)	28.0 ± 4.1
Pre-pregnancy BMI (kg.m^−2^)	22.8 ± 3.5
Neck circumference (cm)	33.8 ± 2.4

### Sleep parameters

The sleep description data are presented in Table 3. Three (10 %) participants did not remember what position they went to sleep in (whom the video recorded as being two right lateral and one supine). Twenty two participants (73 %, Kappa 0.52) accurately recalled the position they went to sleep in. Twenty one participants (70 %) maintained the position at sleep onset for more than 60 min, and only one participant for less than 25 min, with a group median (range) of 73.5 (7.2–261.1) minutes. The wake position could not be determined from video on one occasion when the infrared light failed near the end of the study. The position was determined using the PSG position sensor for this one participant. Six (20 %) participants could not recall what position they woke in. The position at waking was in agreement between video and questionnaire for twelve (40 %) participants (Kappa 0.24). There was no relationship between position at sleep onset and waking (Kappa −0.13).

**Table 3:** Sleep and questionniare summary descriptive data

Variable	Video	Questionnaire
Number who slept on left side of bed	23/30	-
Number where the bed partner was present	16/30	-
Sleep onset position		
Left	20/30	21/30
Right	8/30	4/30
Supine	2/30	4/30
Don’t know	-	3/30
Wake position		
Left	15/30	8/30
Right	11/30	11/30
Supine Prone	4/30 -	4/30 1/30
Don’t know	-	6/30
Times out of bed	1 (0 – 6)	1 (0 – 6)
Total time out of bed (minutes)	2.5 (0 – 21.3)	-
Study duration (hours)	8.6 (6.8 - 9.8)	-
Estimated sleep duration (hours)	8.2 (6.6 – 9.5)	7.0 (4.0 – 9.5)
Estimated sleep latency (minutes)	11.4 (4.5 – 50.6)	20.0 (1.0 – 60.0)
Total time in each position (hours)		
Left	4.3 (0.45 - 7.7)	-
Right	2.0 (0–5.7)	-
Supine	1.6 (0–11)	-
Total time in each position (% of sleep study time)		
Left	49 % (5–90 %)	-
Right	26 % (0–67 %)	-
Supine	19 % (0–75 %)	-
Average time spent in position before changing (minutes)		
Left	34.8 (3.2 – 55.2)	-
Right	39.4 (0 – 58.6)	-
Supine	14.8 (0 – 50.0)	-
Number of times in each position		
Left	4.5 (1 – 10)	-
Right	3 (0 – 7)	-
Supine	3 (0 – 11)	-
Number of position changes	8 (1–22)	
Leg twitches during the sleep study?		
Yes	-	4/30
No	-	13/30
DK	-	13/30
Number of participants that reported difficulty falling asleep	-	22/30
Number of participants that reported difficulty getting back to sleep after waking	-	13/30
Overall self-reported sleep quality in questionnaire		
“Very bad”	-	0/30
“Fairly good”	-	8/30
“Average”	-	16/30
“Fairly bad”	-	6/30
“Very good”	-	0/30
Reported restlessness in questionnaire		
“Not at all”	-	2/30
“A little”	-	7/30
“Average”	-	14/30
“More than average”	-	6/30
“Very restless”	-	1/30

Data presented as number of participants out of sample of 30 participants, or as Median (Range)

There was good agreement between questionnaire data and the video regarding the number of times the women reported getting out of bed and the number of times they were observed getting out of bed (Kappa 0.65). It was not possible to assess leg movements from the video due to the bed clothes. Therefore the ability of participants to recall this could not be verified.

**Fig. 1:**
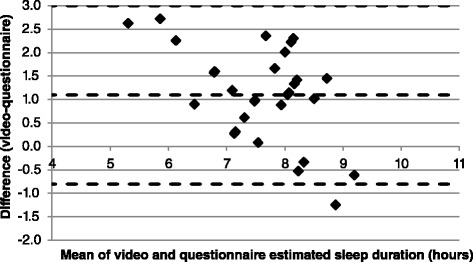
Bland-Altman plot for estimated sleep duration; difference between video and questionnaire. Broken lines indicate mean difference and upper and lower limits of agreement (95 % confidence intervals)

**Fig. 2:**
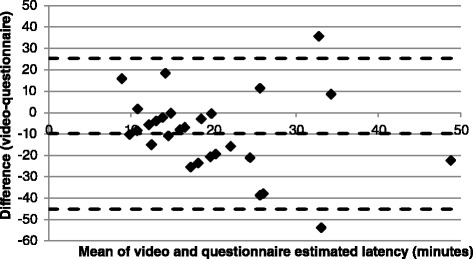
Bland-Altman plot for estimated sleep latency; difference between video and questionnaire. Broken lines indicate mean difference and upper and lower limits of agreement (95 % confidence intervals)

Sleep duration as estimated by the video and in the questionnaire had a correlation of *r*=0.60 (*p*=0.001). The Bland-Altman plot (Fig. 1) demonstrated a mean difference in estimated sleep duration of 1.1 h (95 % CI: −0.8, 3.0 h). There was poor correlation between estimated sleep latency in the video and questionnaire (*r*=−0.18). The Bland-Altman plot (Fig. 2) demonstrated that sleep latency was on average overestimated by 9.8 min (95 % CI: −45.0, 25.5 min). Most participants underestimated sleep duration and overestimated sleep latency (87 % and 80 % of participants, respectively), but both Bland-Altman plots demonstrate negative trends, suggestive of a poor agreement at shorter sleep durations and longer sleep latency, and smaller differences when sleep duration is longer and when sleep latency is short.

According to the automatic snore detection, 25 (83 %) of the participants snored at least some of the night. When asked if they snored on the study night, six (20 %) responded “Yes”, nine (30 %) responded “No”, and 15 (50 %) responded “Don’t know”. There was no clear association between any of the PSG derived snoring statistics and questionnaire response (data available in supplementary files; Additional file 2).

### PSG

The apnoea-hypopnoea index (AHI) and oxygen desaturation index (ODI) derived from the PSG could not be measured in two participants due to failure of the pulse oximetry. The median (range) AHI and ODI for the remaining 28 participants were 0.2 (0 – 7.9) and 0.9 (0 – 36.1) events per hour, respectively. The individuals’ average pulse oximetry over the night had a median (range) of 97 % (95 – 98). One participant was classified as having mild obstructive sleep apnoea, with an AHI of 7.9/h (normal being < 5/h) and an ODI of 14.5/h (normal being < 5/h). One participant was diagnosed with probable high upper airways resistance, and had an ODI of 36.1/h (mean saturation 96.7 %, average desaturation 3.8 %), but an AHI of only 0.2/h.

## Discussion

The purpose of this study was to assess the accuracy of self-reported aspects of a night’s sleep in late pregnancy in healthy women. The primary variables of interest were the position at sleep onset and waking, and the number of times participants got out of bed during the night, as these were the most important modifiable factors related to sleep identified by The Auckland Stillbirth Study [1].

The position at sleep onset was identified at the first time point where the participants lay still for greater than three minutes. The majority of participants were identified as falling asleep on their left side (67 %), and a minority settled to sleep in the supine position (7 %). There was good agreement for sleep onset position between video and questionnaire, and sleep onset position was maintained for greater than an hour in 70 % of participants, with only one participant changing in less than 25 min. In contrast, in only 40 % of participants did video confirm the stated recalled waking position. However, the accuracy of the recalled position at sleep onset and waking may have been better than our data suggests, as there may have been a discrepancy between the video assessment criteria and the self report. This is particularly likely for waking, where “waking up” can occur over time, or in phases, making it difficult for the participant and the video assessor to define when waking occurred. Sleep staging with EEG might have addressed this discrepancy, but ambulatory EEG was not available for this study. However, as with video determined sleep onset, true-physiological sleep onset and waking may still be at a different time from that perceived by the women, especially in fragmented sleep or in a prolonged wake-up period. Unlike The Auckland Stillbirth Studies [1] the position at sleep onset and wake was not related. In The Auckland Stillbirth Study, 27 % of women reported going to sleep on the left side. The increased number in the current study initiating sleep on the left side (67 %) may have contributed to both their improved recall of sleep onset position, and the poorer relationship with position at awakening, as sleep onset position is likely to be deliberate, whereas position at awakening is not. Although it was not asked in the questionnaire, it is possible that the awareness of The Auckland Stillbirth Study findings in this Auckland-based population influenced the intentional adoption of the left lateral position at sleep onset.

The number of times the participants were observed getting out of bed matched the reported number of visits to the toilet in 22 out of the 30 participants (73 %). The Kappa statistic (0.65) indicates that this variable is recalled fairly accurately.

There was good recall of sleep duration. However, as demonstrated by the Bland-Altman plot (Fig. 1), the agreement improved as sleep duration increased. Short sleep duration has been linked to underestimation of sleep duration (although not to objective PSG measures of fragmentation) in patients with insomnia symptoms [14]. However, another study observed no relationship between objectively (EEG) measured total sleep time and subjective reports by third trimester women, with equal numbers overestimating and underestimating total sleep time [15]. In the current study, sleep duration was estimated by lack of movement (and wakening by the ceased immobility) in the video data. This may lead to over estimation of sleep as video assessment cannot accurately assess the amount of time that participants were awake whilst lying still in bed, and thus may have contributed to the underestimation by the participants. Previously reported mean sleep durations of 7.5 ± 1.8 h [10] and 7.45 ± 1.19 h [9] in late pregnancy were less than the median value estimated by the video analysis (8.2 h), but higher than the self-reported values (7.0 h) in this study.

The correlation between the two measures of estimated sleep latency was weak, and the limits of agreement (95 % confidence intervals) were too large to be considered clinically acceptable (±35 min). Consistent with the current study, a previous study showed that approximately 80 % of both non-pregnant and third-trimester women overestimate sleep latency when compared to objectively (EEG) measured sleep latency [15]. However, Wilson et al. [15] also demonstrated that the definition used for sleep latency affects agreement, with sleep latency to the first ten minutes of continuous sleep having the greatest agreement with perceived sleep latency in the pregnant group. The first period of three minutes of immobility was used in the current study, but perhaps the time to the first ten minutes of continuous sleep measured objectively using EEG would have yielded greater agreement. Regardless, both this study and that of Wilson et al. [15] demonstrate that sleep latency is difficult to estimate.

Self-reported snoring is a measure commonly used in sleep research in pregnancy, but has inconsistent findings regarding its association with pregnancy outcomes e.g. [16–21]. The derived snoring statistics in the current study suggest that self-reported snoring may not be a valid measure in healthy pregnancy where the snoring may not be considered severe, which may explain the inconsistency regarding pregnancy outcomes. It is important to note that the snoring statistics were calculated from the derived snoring signal, determined by vibrations in the nasal cannula. This may detect very quiet snoring or flow limitation that would not disturb the participant or their bed partner, and which may or may not be clinically important.

This study provides additional information to the pregnancy sleep literature. The participants in the study of Warland and Dorrian [9] kept a sleep diary, and women were instructed to sleep on their left side. Their study demonstrated that pregnant women were able to estimate the time spent in different positions with moderate accuracy, and they could change the position they slept in when instructed to do so. The efficacy of such interventions will provide important information if it is confirmed that a certain sleep position can be protective against adverse events without meaningful reductions in sleep quality. The current study tested the recall of a night’s sleep in late pregnancy, with no instruction on how to sleep, nor were participants made more aware of their sleep behaviour by keeping sleep diaries or having knowledge of the questionnaire content. These studies together with that by O’Brien [8] provide some very useful and encouraging information. Collectively, they show that women can accurately recall the positions they adopt at sleep onset, and to a lesser extent, at waking, and provide a description of sleep, whether as a “usual” night or one where they are instructed to change their habits [9].

A limitation of our study was that, unlike Warland and Dorrian [9], we did not ask women what proportion of the night they believed they spent in each position. Whilst position at sleep onset and waking was shown to be associated with late stillbirth risk in The Auckland Stillbirth Study, sleep position at onset and wake may or may not correlate with sleep positions adopted by pregnant women for the majority of the night. This may have been a useful measure to include in the questionnaire in this study.

As only a level III PSG device (non-EEG) was available for this study, we were unable to assess sleep staging and identify true physiological sleep onset (and therefore sleep latency) and waking. The use of EEG may have facilitated agreement for position at sleep onset and wakening, and perhaps based on the recommendations of Wilson et al. [15] also sleep latency. Surface electromyography would have been useful to detect leg movements and to assess if the women were aware of its occurrence.

## Conclusions

This study provides useful information about patterns of normal sleep in healthy late pregnancy, and demonstrates the accuracy of certain self-reported aspects of sleep behaviours. Sleep-related factors that have been associated with late still birth incidence [1], including position at sleep onset and number of times the women got out of bed, were found to be recalled accurately. Therefore, self-reported measures of these variables can be considered reliable. Position at waking was found to have a lower accuracy, although this may be due to methodological issues, or the effects of a phased or prolonged wake time.

Future studies may consider a Level I (in-lab) or Level II (unattended) PSG with EEG for sleep staging. Future studies could examine how sleep patterns differs in obese or high-risk pregnant women, in non-singleton pregnancies, or in the presence of confirmed sleep disordered breathing in pregnancy, as well as the ability to recall events accurately.

## Ethics approval

The Northern X Regional Human Ethics Committee approved the study protocol (NTX/12/06/048), and all participants provided written informed consent.

## Consent for publication

Not applicable.

## Availability of data and materials

The datasets supporting the conclusions of this article are included within the manuscript (and its additional files). Written permission to publish indirect identifiers (e.g. age, BMI, gestation, fetal descriptors) was not obtained and to protect participants’ identity, will not be shared. Raw de-identified sleep data can be provided upon request.

## Additional files

Additional file 1: Sleep questionnaire. (DOCX 83 kb) Additional file 2: Snoring statistics by questionnaire snoring response. (DOCX 15 kb)

## Abbreviations

AHI: Apnoea-hypopnoea index
BMI: Body mass index
EEG: Electroencephalography
ODI: Oxygen desaturation index
PSG: Polysomnography (sleep study)
