# Supplementary material for: A description of sleep behaviour in healthy late pregnancy, and the accuracy of self-reports
Source: BMC Pregnancy Childbirth. 2016 May 18;16:115. doi: 10.1186/s12884-016-0905-0 (PMC4870756; doi:10.1186/s12884-016-0905-0)
Supplement: Additional file 1: — Sleep questionnaire. (DOCX 83 kb) [file 12884_2016_905_MOESM1_ESM.docx]

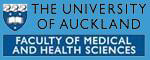

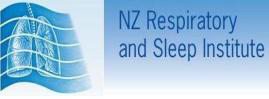


**Maternal Sleep in Pregnancy Questionnaire**

*The following questions relate to your sleep last night:*

# Study ID

| **1. Last night, how long do you think it took you to fall asleep?**  _ _ _ __ (minutes) |  | | | | |
| --- | --- | --- | --- | --- | --- |
|  |  | | |  | |
| **2. Did you have difficulty falling asleep?** 🞏 yes 🞏 no |  | | | | |
|  |  | | |  | |
| **3. How many hours of actual sleep do you think you got?** _ _ _ __ (h) _ _ _ _ (m) |  | | | | |
|  |  | | |  | |
| **4. Did you wake up during the night?** 🞏 yes 🞏 no  If YES, how many times? _ _ _ __ |  | |  | | |
|  |  | |  | | |
| **5. If you woke up in the night did you go to the toilet?** 🞏 yes 🞏 no  If YES, how many times? _ _ _ __ |  | |  | | |
|  |  | | |  | |
| **6. If you woke did you have difficulty getting back to sleep?** 🞏 yes 🞏 no 🞏 didn’t wake |  | | | | |
|  |  | | |  |  |
| **7. What position did you fall asleep in last night?**  🞏 Left side 🞏 Back 🞏 Right side 🞏 Front 🞏 Don’t remember |  | | | | |
|  |  |  | | | |
| **8. What position did you wake up in?**  🞏 Left side 🞏 Back 🞏 Right side 🞏 Front 🞏 Don’t remember |  | | | | |
|  |  | | |  | |
| **9. Did you change sleep position during the night?**  🞏 Not at all 🞏 possibly once 🞏 possibly twice 🞏 More than twice but not lots 🞏 Lots of times |  | | | | |
|  |  | | |  | |
| **10. Would you describe yourself as a restless sleeper last night (i.e. move a lot during the night)?**  🞏 Not at all 🞏 A little 🞏 Average 🞏 More than average 🞏 Very restless |  | | | | |
|  |  | | |  | |
| **11. Did you snore last night?** 🞏 yes 🞏 no 🞏 don’t know |  | | | | |
|  |  | | |  | |
| **12. Did your legs twitch or jerk often while you slept last night?** 🞏 yes 🞏 no 🞏 don’t know |  | | | | |
|  |  | | |  | |
| **13. Did you take medication for sleep last night?** 🞏 yes 🞏 no |  | | | | |
|  |  | | |  | |
| **14. Overall, how would you rate your sleep quality last night?**  🞏 Very good 🞏 Fairly good 🞏 Average 🞏 Fairly bad 🞏 Very bad |  | | | | |
|  |  | | |  | |
| **15. Which ethnic group do you belong to?** *Mark the space or spaces that apply to you.*  🞏 NZ European 🞏 Maori 🞏 Samoan 🞏 Cook Island Maori 🞏 Tongan 🞏 Niuean 🞏 Chinese 🞏 Indian 🞏 Other (*such as Dutch, Japanese, Tokelauan).* Please state: _________________ |  | | | | |
|  |  | | |  | |
| **16. Number of other children (exclude this pregnancy)** |  | | | | |
| **17. Date baby is due _____________________________**  **Today’s date**  **_____________________________** | | | | |  |

*Thank you for your participation. It was much appreciated.*
