# Supplementary material for: A description of sleep behaviour in healthy late pregnancy, and the accuracy of self-reports
Source: BMC Pregnancy Childbirth. 2016 May 18;16:115. doi: 10.1186/s12884-016-0905-0 (PMC4870756; doi:10.1186/s12884-016-0905-0)
Supplement: Additional file 2: — Snoring statistics by questionnaire snoring response. (DOCX 15 kb) [file 12884_2016_905_MOESM2_ESM.docx]

**Additional file 2: Snoring statistics by questionnaire snoring response**

| Snoring statistic | Responded “Yes”  N = 6 | Responded “No”  N = 9 | Responded “Don’t know”  N = 15 |
| --- | --- | --- | --- |
| Total snore time (minutes) | 55 (4 – 81) | 89 (0 – 340) | 41 (3 – 123) |
| Proportion of study spent snoring (%) | 11 (0 - 16) | 22 (0 - 69) | 7 (1 - 27) |
| Total number of snoring episodes | 94 (14 – 133) | 42 (1 – 255) | 96 (9 - 182) |
| Average duration of snoring episode (minutes) | 0.55 (0.30 - 0.80) | 0.50 (0.05 – 1.10) | 0.40 (0.30 – 0.50) |
| Longest snoring episode (minutes) | 4 (1 - 7) | 2 (0 - 18) | 3 (0 - 5) |

Presented as median (IQR)
